# Kagome superconductors AV_3_Sb_5_ (A = K, Rb, Cs)

**DOI:** 10.1093/nsr/nwac199

**Published:** 2022-09-27

**Authors:** Kun Jiang, Tao Wu, Jia-Xin Yin, Zhenyu Wang, M Zahid Hasan, Stephen D Wilson, Xianhui Chen, Jiangping Hu

**Affiliations:** Beijing National Laboratory for Condensed Matter Physics and Institute of Physics, Chinese Academy of Sciences, Beijing 100190, China; School of Physical Sciences, University of Chinese Academy of Sciences, Beijing 100190, China; Hefei National Laboratory for Physical Sciences at the Microscale, University of Science and Technology of China, Hefei 230026, China; CAS Key Laboratory of Strongly-coupled Quantum Matter Physics, Department of Physics, University of Science and Technology of China, Hefei 230026, China; Laboratory for Quantum Emergence, Department of Physics, Southern University of Science and Technology, Shenzhen 518055, China; CAS Key Laboratory of Strongly-coupled Quantum Matter Physics, Department of Physics, University of Science and Technology of China, Hefei 230026, China; Laboratory for Topological Quantum Matter and Advanced Spectroscopy (B7), Department of Physics, Princeton University, Princeton, NJ 08544, USA; Materials Department and California Nanosystems Institute, University of California Santa Barbara, Santa Barbara, CA 93106, USA; Hefei National Laboratory for Physical Sciences at the Microscale, University of Science and Technology of China, Hefei 230026, China; CAS Key Laboratory of Strongly-coupled Quantum Matter Physics, Department of Physics, University of Science and Technology of China, Hefei 230026, China; Beijing National Laboratory for Condensed Matter Physics and Institute of Physics, Chinese Academy of Sciences, Beijing 100190, China; Kavli Institute of Theoretical Sciences, University of Chinese Academy of Sciences, Beijing 100190, China

**Keywords:** kagome superconductor, charge density wave, time-reversal symmetry breaking, topological metal

## Abstract

The quasi-two-dimensional kagome materials AV_3_Sb_5_ (A = K, Rb, Cs) were found to be a prime example of kagome superconductors, a new quantum platform to investigate the interplay between electron correlation effects, topology and geometric frustration. In this review, we report recent progress on the experimental and theoretical studies of AV_3_Sb_5_ and provide a broad picture of this fast-developing field in order to stimulate an expanded search for unconventional kagome superconductors. We review the electronic properties of AV_3_Sb_5_, the experimental measurements of the charge density wave state, evidence of time-reversal symmetry breaking and other potential hidden symmetry breaking in these materials. A variety of theoretical proposals and models that address the nature of the time-reversal symmetry breaking are discussed. Finally, we review the superconducting properties of AV_3_Sb_5_, especially the potential pairing symmetries and the interplay between superconductivity and the charge density wave state.

## Teaser

In this review, the authors report recent progress on the experimental and theoretical studies of kagome superconductor AV_3_Sb_5_, which opens a new route towards unconventional orders in kagome metals.

## INTRODUCTION

**Figure 1.**
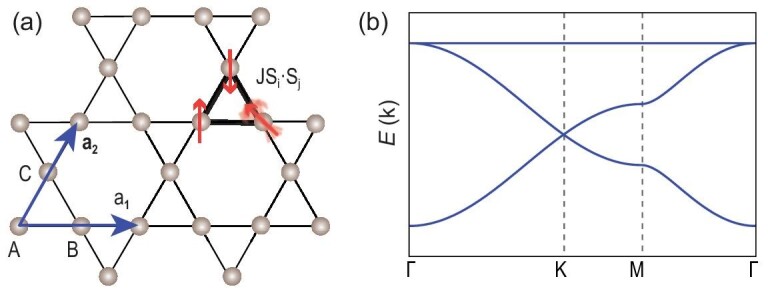
(a) The crystal structure for the kagome lattice, which originated from a Japanese basket-weaving pattern. The translation vectors are labeled $\mathbf {a}_1$ and $\mathbf {a}_2$. In each unit cell, there are three sublattices, labeled A, B, C. For the nearest-neighbor Heisenberg model $\mathbf {J} \mathbf {S}_i \cdot \mathbf {S}_j$, the kagome lattice faces geometric frustration. As illustrated in the upper corner, if two adjacent spins are set antiparallel, the third spin will face a dilemma. (b) Band structures for the nearest-neighbor tight-binding model on the kagome lattice.

Unveiling new physics from simple lattice models plays a vital role in modern condensed matter physics. For instance, the exact solution of the two-dimensional (2D) Ising model on a square lattice by Onsager revolutionized our view of phase transitions in statistical physics [1,2]; honeycomb lattice of graphene can be used to mimic the physics of quantum electrodynamics for Dirac fermions [3–5]. Motivated by Onsager’s solution [1], the kagome lattice was introduced to statistical physics by Syozi [6], which serves as a rich lattice for realizing novel states and phase behaviors [7–11]. As shown in Fig. 1(a), a kagome lattice is formed by corner-sharing triangles. There are three sublattices labeled A, B, C, inside each triangle forming the unit cell. Owing to this special lattice structure, the kagome lattice contains geometric frustration for spin systems, which gives rise to extensively degenerate ground states in the nearest-neighbor antiferromagnetic Heisenberg model [12], as illustrated in Fig. 1(a). Accordingly, the ground state of the kagome spin model is the most promising candidate for the long-sought quantum spin liquid states [8–10].

Recently, fermionic models on kagome lattices have also become an important platform for studying the interplay among electron-electron correlation effects, band topology and lattice geometry [13]. The point group of the kagome lattice is the same as graphene [3], and a standard nearest-neighbor tight-binding model on the kagome lattice exhibits Dirac cones at K points, as shown in Fig. 1(b). Many distinct properties associated with Dirac fermions [3] have been discussed, including the $\sqrt{n B}$ Landau level [14], tunable Dirac gaps [15,16], Chern gaps [14] and the quantum anomalous Hall effect [17,18], etc. Besides its Dirac cones, a kagome lattice model can also display flat bands, as shown in Fig. 1(b). The flat band arises from the destructive quantum interference of the wave functions from each of the three sublattices. Studying exotic phenomena on flat bands, like fractional Chern insulator states, has been carried out both theoretically and experimentally [19–25].

In addition to these phenomena, superconductivity in kagome lattice materials has also been widely discussed. It has been argued that the kagome lattice can host a variety of unconventional pairing superconducting states, including the *d* + *id* chiral superconductor (SC) [26–28] and *f*-wave spin-triplet SC [29], among others. However, superconducting kagome materials are rare in nature. Last year, the newly discovered kagome material CsV_3_Sb_5_ [30] was found to be a quasi-2D kagome SC with a transition temperature *T_c_* ≈ 2.3 K [31]. Subsequently, superconductivity was also found across the entire family of compounds KV_3_Sb_5_ (*T_c_* ≈ 0.93 K) [32] and RbV_3_Sb_5_ (*T_c_* ≈ 0.75 K) [33]. This discovery has stimulated extensive research activity in this field [30–39].

In this review, we discuss the recent progress in studying this newly discovered AV_3_Sb_5_ kagome family. This paper is organized as follows. We first discuss the crystal structure and the electronic properties of AV_3_Sb_5_ (A = K, Rb, Cs). Second, we review both the experimental evidence and theoretical understanding of the unconventional charge density wave order that forms and reports of accompanying time reversal symmetry breaking. Third, we report the current status of understanding the SC properties of AV_3_Sb_5_. Finally, we address other unconventional features in these compounds, such as pairing density wave order, and provide future research perspectives.

## CRYSTAL AND ELECTRONIC STRUCTURES

**Figure 2.**
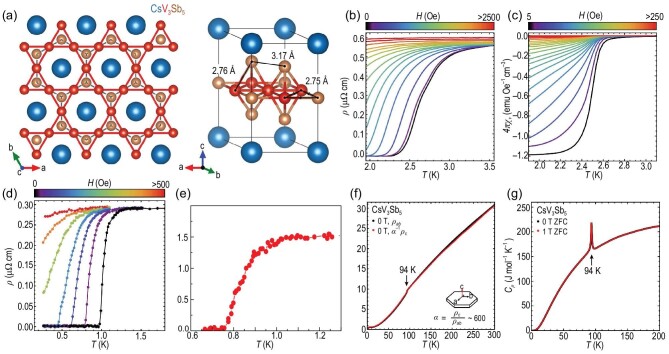
(a) The crystal structure for CsV_3_Sb_5_. Adapted from [31]. (b) and (c) Field-dependent resistivity and magnetization at low temperatures, showing the onset of superconductivity for CsV_3_Sb_5_ with *T_c_* ≈ 2.3 K. Adapted from [31]. (d) Field-dependent resistivity at low temperatures for KV_3_Sb_5_. Adapted from [32]. (e) Resistivity at low temperatures for RbV_3_Sb_5_. Adapted from [33]. (f) and (g) The temperature-dependent electrical resistivity, and heat capacity (zero field cooled (ZFC)) at higher temperature for CsV_3_Sb_5_ showing a transition around 94 K. Adapted from [31].

The AV_3_Sb_5_ materials crystallize into the *P*6/*mmm* space group and exhibit a layered structure of V-Sb sheets intercalated by K/Rb/Cs, as shown in Fig. 2(a) and (b) [30]. In the V-Sb plane, three V atoms form the kagome lattice and an additional Sb atom forms a triangle lattice located at the V kagome lattice’s hexagonal center. This V kagome layer largely dominates the physics behind AV_3_Sb_5_, as discussed later. Above and below the V-Sb plane, out-of-plane Sb atoms form two honeycomb lattice planes respectively with lattice sites located above and below the centers of the V triangles in the kagome plane. A-site atoms form another triangular lattice above or below these Sb honeycomb or antimonene planes.

We can first understand the electronic properties of AV_3_Sb_5_ from the transport measurements. The low-temperature electrical resistivity ρ(*T*) and its field dependence are plotted in Fig. 2(b) for CsV_3_Sb_5_ [31]. One finds that the zero field ρ(*T*) shows a broad transition towards the SC ground state with *T_c_* ≈ 2.3 K, which is continuously suppressed by applying a magnetic field. The magnetization data in Fig. 2(c) also reveals a well-defined Meissner effect, and heat capacity measurements show a sharp entropy anomaly at the SC transition [31]. Therefore, the CsV_3_Sb_5_ becomes the first example of quasi-2D kagome SCs. The critical field *H_c_* for CsV_3_Sb_5_ is relatively small with the *c* direction *H*_*c*2_ ≈ 0.4T [40,41]. Similarly, the ρ(*T*) of KV_3_Sb_5_ drops to zero with *T_c_* ≈ 0.93 K shown in Fig. 2(e) [32] and RbV_3_Sb_5_ has a *T_c_* ≈ 0.75 K [33]. Hence, all AV_3_Sb_5_ compounds within the material family are superconducting at low temperature.

Above the SC ground state, the normal states of AV_3_Sb_5_ also show quite different behavior. The temperature-dependent resistivity of KV_3_Sb_5_ can be modeled by a Fermi-liquid formula ρ(*T*) = ρ_0_ + *aT*^2^ [30], which shows a typical metallic behavior. The in-plane and out-plane resistivity data show a large anisotropy with a ratio α = ρ_*c*_/ρ_*ab*_ ≈ 600 in CsV_3_Sb_5_, as shown in Fig. 2(f) [31]. This large anisotropy agrees well with the quasi-2D nature of AV_3_Sb_5_, where the V kagome layers play a dominant role in the electronic properties. Hence, the AV_3_Sb_5_ is a quasi-2D metal. The resistivity ρ(*T*) also contains a kink behavior around 94 K, which is related to the long-range charge-density wave (CDW) order discussed later [31]. A sharp peak from the heat capacity data at this same temperature indicates that the CDW transition is a first-order phase transition [31], where the first derivatives of free energy are not continuous. The lack of phonon softening near this transition from the inelastic x-ray scattering also suggests that the transition is weakly first order [42,43]. It is worth mentioning that this weak first-order transition is best characterized in CsV_3_Sb_5_, and the nature of the transition merits further study in other compounds.

**Figure 3.**
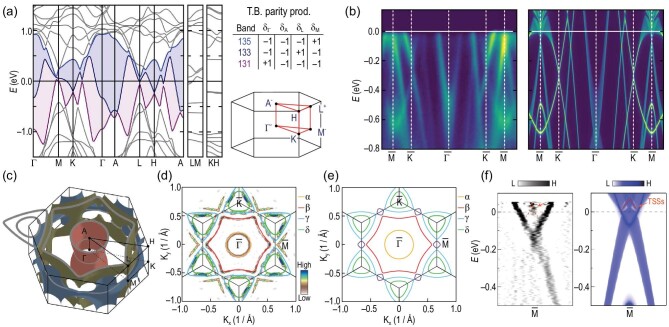
(a) The band structure of CsV_3_Sb_5_ calculated by DFT. The insert shows the parity eigenvalues for each band at the time-reversal invariant momentum points. Adapted from [31]. (b) ARPES measured band structure (left) and its comparison with DFT (right) for CsV_3_Sb_5_. Adapted from [31]. (c) FS calculated for CsV_3_Sb_5_ at experimental *E_F_*. Adapted from [52]. (d) and (e) FSs measured by ARPES and calculated by DFT for KV_3_Sb_5_. Adapted from [51]. (f) The ARPES measured (left) and DFT-calculated (right) topological surface states (TSSs) for CsV_3_Sb_5_. Adapted from [48].

To reveal the electronic nature of AV_3_Sb_5_, density functional (DFT) calculations and angle-resolved photoemission spectroscopy (ARPES) measurements have been performed [30,31,42,44–53]. The DFT calculations show multiple bands crossing the Fermi level (*E_F_*) in CsV_3_Sb_5_, as shown in Fig. 3(a). Around the Γ point, there is an electron-like parabolic band, which originates from the in-plane Sb *p_z_* orbital. The bands around the Brillouin zone (BZ) boundaries are mainly attributed to the V *d* orbitals. Note that there are two van Hove (VH) points close to *E_F_* around the M point, which play an important role in the symmetry breaking observed in AV_3_Sb_5_. The upper VH point is further connected with the Dirac cone around the K point, which reflects a typical feature of the kagome model described above. ARPES measurements show that the electronic band structure of CsV_3_Sb_5_ qualitatively agrees with DFT calculations [31], as shown in Fig. 3(b), and DFT calculations provide qualitatively accurate descriptions of the electronic structures of AV_3_Sb_5_ systems. Note that there are still discrepancies between quantum oscillations and DFT calculations [52,54], which calls for future studies.

To confirm the quasi-2D nature of AV_3_Sb_5_, the three-dimensional Fermi surface (FS) of CsV_3_Sb_5_ is calculated in Fig. 3(c). The FSs show the traditional cylinder behaviors as in copper-based and iron-based superconductors [55–57], which is the origin of large resistivity anisotropy. The excellent agreement between DFT and ARPES indicates a small band renormalization owing to correlation effects in the lattice. Hence, the AV_3_Sb_5_ materials are effectively modeled as weakly correlated systems [58]. For example, the high-resolution ARPES data from KV_3_Sb_5_ find excellent matching between the measured and calculated FSs [51], as plotted in Fig. 3(d) and (e).

Besides the above electronic structures, CsV_3_Sb_5_ also carries a non-trivial *Z*_2_ topological index [31]. For inversion symmetric and time-reversal symmetric systems, the *Z*_2_ topological invariant can be obtained from time-reversal invariant momentum points with their inversion operator eigenvalues [59]. As listed in Fig. 3(a), the *Z*_2_ invariant is non-trivial for band numbers 131, 133, 135 enumerated in DFT calculations. The parity index for 133, 135 bands at the M point is different, which gives rise to a band inversion at M. Therefore, the normal state of CsV_3_Sb_5_ is a *Z*_2_ topological metal, and this *Z*_2_ topological property leads to a surface state embedding around the bulk FS at the M point. ARPES experiments have resolved this feature, as shown in Fig. 3(f).

## CHARGE-DENSITY WAVE AND SYMMETRY BREAKING

**Figure 4.**
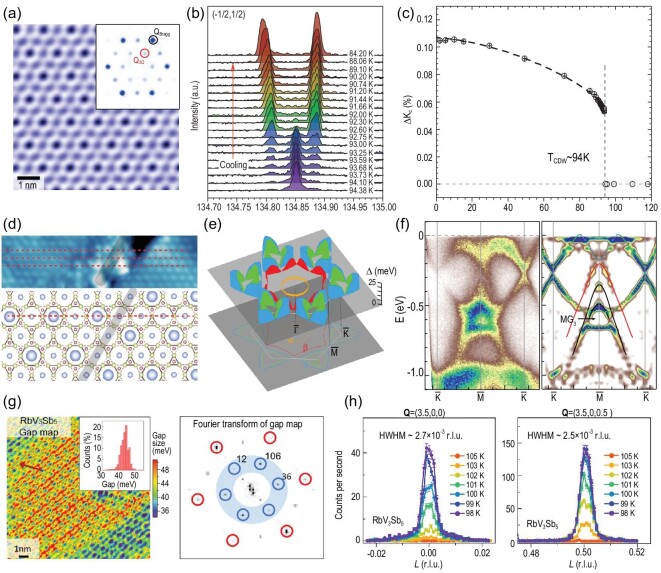
(a) A topographic image of a large Sb surface and its Fourier transformation showing a 2 × 2 modulation for KV_3_Sb_5_ from STM. Besides the Bragg peaks *Q*_Bragg_, there are additional charge modulation peaks *Q*_3Q_. Adapted from [38]. (b) The temperature dependence of the central transition lines of ^51^V NMR with the temperature cooling across *T*_CDW_ for CsV_3_Sb_5_. Adapted from [67]. (c) Temperature dependence of the splitting of Knight shift Δ*K_c_* for CsV_3_Sb_5_. Adapted from [67]. (d) The STM scanning of the step edge in CsV_3_Sb_5_. The dashed lines track the chains with CDW modulation on the upper side. A π-phase jump can be observed between the upper and lower sides. The illustration of the CDW patterns near a single-unit-cell step is plotted in the lower panel. Adapted from [62]. (e) The CDW gap structures for each FSs in KV_3_Sb_5_ measured by ARPES. Adapted from [51]. (f) ARPES measured band structures (right) and their second derivatives along $\bar{K}$-$\bar{M}$-$\bar{K}$. There is one additional gap MG_3_ away from *E_F_*. Adapted from [51]. (g) Real-space CDW gap map for RbV_3_Sb_5_ and its Fourier transform. The 2 × 2 vector peaks show different intensities, defining a kind of electronic chirality. Adapted from [66]. (h) The temperature-dependent CDW peaks of RbV_3_Sb_5_ at *Q* = (3.5, 0, 0) and (3.5, 0, 0.5). The CDW peak at half-integer L demonstrates a 3D CDW with 2 × 2 × 2 superstructure. Adapted from [42].

As discussed in the previous section, a CDW phase transition occurs for all AV_3_Sb_5_ materials ranging from 78 to 103 K (*T*_CDW_ ≈ 94 K for CsV_3_Sb_5_, *T*_CDW_ ≈ 103 K for RbV_3_Sb_5_, *T*_CDW_ ≈ 78 K for KV_3_Sb_5_) [30–33]. In the first report of AV_3_Sb_5_ crystal growth, elastic neutron scattering measurements ruled out the possibility of long-range magnetic order [30]. The absence of long-range magnetic order was further confirmed by the muon spin spectroscopy, indicating the transition derives primarily from the charge degree of freedom [37]. Soon after SC was discovered in CsV_3_Sb_5_, scanning tunneling microscopy (STM) measurements were performed on the Sb and K surfaces of KV_3_Sb_5_, revealing that the transition is a CDW transition with 2 × 2 superlattice modulation [38,60–66]. From the STM topographic spectrum in Fig. 4(a), the charge modulation on the Sb surface is resolved [38]. By Fourier transforming the topographic image, there are six additional ordering peaks Q_3Q_ in addition to those from the primary lattice structure [38]. STM further shows an energy gap opened around the Fermi energy of ∼50 meV, which together with the 2 × 2 superlattice modulation disappears above *T*_CDW_ [38,60–66]. Across this gap, there is a real-space charge reversal for the 2 × 2 superlattice modulation [38], which is a hallmark of CDW ordering.

Nuclear magnetic resonance (NMR) measurements further support the absence of magnetic order and confirm that the CDW transition is indeed a first-order transition [67]. From the NMR spectrum, there are two V signals after the CDW transition, V(I) and V(II), as shown in the inset of Fig. 4(b). The splitting of Knight shift Δ*K_c_* between V(I) and V(II) sites shows a sudden jump at *T*_CDW_. Beyond the surface sensitive measurements, the CDW state is found to be three dimensional and be modulated along the *c* axis. This modulation is either 2 × 2 × 2 or 2 × 2 × 4 for AV_3_Sb_5_ materials with 2 × 2 × 2 reported for KV_3_Sb_5_ and both 2 × 2 × 2 and 2 × 2 × 4 reported for CsV_3_Sb_5_ [38,42,43,52], as shown in Fig. 4(g) and (h). Disorder along the *c* axis impacts crystallinity in the direction of the out-of-plane modulation and potentially accounts for this discrepancy. The 3D modulation is also confirmed by the STM data collected across surface step edges [62] and a ^133^*Cs* NMR spectrum study [67]. Future studies are underway to fully understand the *c*-axis periodicity of the superlattice. On the clean surface regions of CsV_3_Sb_5_ and RbV_3_Sb_5_, STM detects real-space modulations of the CDW gap, as shown in Fig. 4(g). Interestingly, the Fourier transform of the gap map also shows the 2 × 2 vector peaks with different intensities, thus revealing a novel electronic chirality of the CDW order [38,66].

In order to determine the gap structures in momentum space, several high-resolution ARPES measurements have been performed [42,49–51,53]. Based on ARPES data, we can find that different FSs in KV_3_Sb_5_ exhibit diverse CDW gap structures, as shown in Fig. 4(e). The CDW gap vanishes for the α FS around the BZ Γ point. Since the α FS stems from the *p_z_* band of the in-plane Sb, the *p_z_* orbital does not participate in the CDW formation [50,51]. In contrast, the V-derived FSs around the BZ boundary exhibit highly momentum-dependent CDW gaps, which are dominated by quasiparticles around the van Hove singularities at the M points [50,51]. Quantum oscillation measurements also support the dominant role of vanadium orbitals within the CDW order [52]. Hence, the V kagome layer dominates the CDW gaps and the VH quasiparticles deeply influence the gap structure in AV_3_Sb_5_. In addition to the gaps resolved around the FSs, ARPES data in KV_3_Sb_5_ have also observed a large CDW gap opening away from *E_F_* [51]. For instance, at the M point, a 125-meV gap opens at MG_3_ at 20 K, as shown in Fig. 4(f). This feature strongly indicates that the structural transition plays an important role in this CDW transition. It is also clear that the structural transition mostly affects the V kagome network, while the out-of-plane coupling involving Sb *p_z_* orbitals is hardly changed.

### Time-reversal symmetry breaking

**Figure 5.**
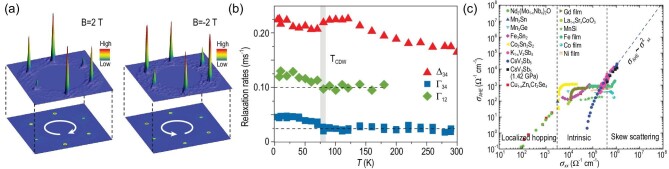
(a) Spectroscopic 2 × 2 vector peaks for KV_3_Sb_5_ taken at B = 2 T and B = −2 T. The highest vector peaks shift their positions under magnetic field. Adapted from [38]. (b) The temperature-dependent muon relaxation rates in KV_3_Sb_5_. The Γ_12_ measures the rates collected in the forward and backward detectors, while the Γ_34_ and Δ_12_ measure the rates collected in the up and down detectors. The relaxation rates start to increase below the CDW transition. Adapted from [69]. (c) Plot of σ_AHE_ versus σ_*xx*_ for a variety of materials compared with CsV_3_Sb_5_ spanning various regimes from the localized hopping regime to the skew scattering regime. Adapted from [39].

Interestingly, accumulated evidence for time-reversal symmetry breaking (TRSB) signals was found in the CDW phases of AV_3_Sb_5_ compounds. Since charge is a quantity to preserve time-reversal symmetry, the emergence of this TRSB becomes one of the more intriguing phenomena in these otherwise non-magnetic AV_3_Sb_5_ materials. The first evidence for TRSB was found in magnetic-field-dependent STM measurements [38]. As discussed above, there are six CDW ordering vectors Q_3Q_ from the STM topographic spectrum. However, the intensities of these three pairs of vectors are different in the clean regions for all AV_3_Sb_5_ materials [38,61,66], thus defining a chirality of the CDW order (counting direction from the lowest intensity peak pairs to highest intensity peak pairs). The chirality of the CDW order further shows an unusual response to the perturbation of external magnetic field B. As shown in Fig. 5(a), the chirality switch from anticlockwise to clockwise when the magnetic fields changes from +2 to −2 T applied along the *c* axis. Owing to the Onsager reciprocal relation, the response functions of a time-reversal preserving system under +*B* and −*B* must relate to each other by a time-reversal operator. This non-reciprocal relation under magnetic field breaks the Onsager relation indicating the TRSB in this non-magnetic kagome system [38]. However, we still want to mention that this chirality signal is missing in recent STM and spin-polarized STM reports [63,68], which deserves further investigations.

The straightforward evidence for TRSB comes from the zero-field muon spin relaxation/rotation (μSR) spectroscopy [69,70]. The spin-polarized muons were implanted into the AV_3_Sb_5_ single crystals. The muon spin will rotate and relax under the influence of local magnetic fields. The μSR technique is highly sensitive to the extremely small magnetic fields, capable of detecting of the order of 0.1 Gauss fields experienced by the implanted muons. As shown in Fig. 5(b), the relaxation rates of KV_3_Sb_5_ start to increase below the CDW transition temperature *T*_CDW_, which strongly suggests the emergence of a local magnetic field owing to TRSB [69]. Similar measurements on CsV_3_Sb_5_ also found TRSB signals [70]. However, the TRSB transition temperature is slightly lower than *T*_CDW_ ≈ 90 K. We return to discuss the physical origin of this TRSB in the next section.

Moreover, a giant anomalous Hall effect (AHE) has also been observed in AV_3_Sb_5_ [35,39], and the onset of this AHE was found to be concurrent with the CDW order [39]. Normally, there are two origins of the AHE: intrinsic Berry curvature and extrinsic impurity scattering [71]. As shown in Fig. 5(c), by comparing transverse σ_AHE_ and longitudinal σ_*xx*_ conductivity, both the intrinsic Berry curvature and the impurity-induced skew scattering contribute to the giant AHE in KV_3_Sb_5_ and CsV_3_Sb_5_. However, compared to conventional spontaneous AHE with ferro- or ferrimagnetic ordering, the AHE in AV_3_Sb_5_ exhibits σ_AHE_(*B* → 0) = 0 without a hysteresis behavior. The σ_AHE_(*B* → 0) = 0 feature might originate from the anti-phase TRSB between adjoining kagome layers or domain walls [70]. The origin of this non-hysteresis anomalous Hall effect is still unclear, which deserves further careful study.

We want to emphasize that the conclusive proof of TRSB in AV_3_Sb_5_ is still lacking. Besides the above μSR, and magnetic-field-dependent STM measurements, results from other TRSB sensitive techniques like the polarized neutron diffraction and Kerr effect are highly desired.

### Spatial symmetries

Besides the translation symmetry breaking and time-reversal symmetry breaking associated with the CDW state, an interesting question is: what are the remaining symmetries within the CDW state? The point group of the AV_3_Sb_5_*P*6/*mmm* space group is *D*_6*h*_, which can be generated by the *C*_6_ rotation, inversion operator ${\cal I}$ and the mirror operator σ_*x*_ about the *y*–*z* plane [72]. Although there is still some debate on what kind of spatial symmetry is broken at low temperatures, knowledge of these generators provides a general outlook of the remaining symmetries.

To test the inversion symmetry ${\cal I}$, second-harmonic generation (SHG) optical data were collected for CsV_3_Sb_5_ [70]. SHG measures the second-order non-linear optical response $\mathbf {P}= \epsilon _0\chi ^{(2)} \mathbf {E} \mathbf {E}$, where $\mathbf {P}$ is the electric polarization induced by the incident light with electric field $\mathbf {E}$ and ε_0_ is the vacuum permittivity. Since $\mathbf {P}$ and $\mathbf {E}$ are odd under inversion symmetry ${\cal I}$, the rank-three non-linear optical susceptibility tensor χ^(2)^ is only finite when parity is broken. Only negligibly small SHG signals (likely originating from the surface) were detected from 120 K down to 6 K. Hence, inversion symmetry ${\cal I}$ remains a valid symmetry for AV_3_Sb_5_ at all temperatures, which constrains the CDW order and will also be important for the superconducting pairing possibilities discussed in the following section.

**Figure 6.**
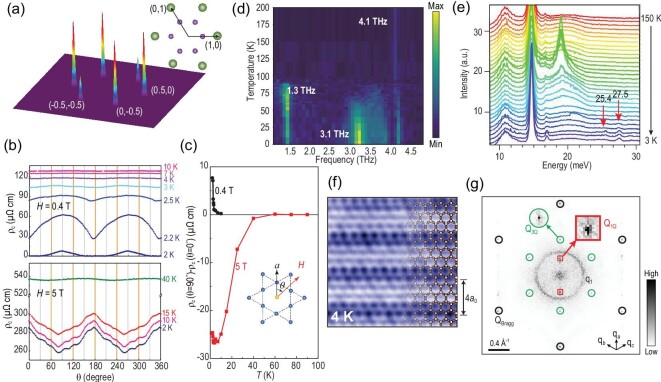
(a) Spectroscopic 2 × 2 vector peaks for KV_3_Sb_5_ taken at zero external field. Adapted from [38,43]. (b) Angular dependent *c*-axis resistivity for CsV_3_Sb_5_ measured at different temperatures under magnetic fields of 0.4 T (upper panel) and 5 T (lower panel). Adapted from [40]. (c) Temperature dependence of nematicity of *c*-axis resistivity between θ = 0° and 90°. Adapted from [40]. (d) Temperature dependence waterfall map of coherent phonon spectroscopy for CsV_3_Sb_5_. Adapted from [76]. The 4.1-THz coherent phonon is present at all temperatures through phase change. The 1.3-THz phonon can only be detected below *T*_CDW_, while the 3.1-THz phonon only shows up at temperatures below 30 K–60 K. (e) Raman spectroscopy for KV_3_Sb_5_. Below 30 K, two new phonon modes at 25.4 and 27.5 meV are observed. Adapted from [42]. (f) and (g) The 1 × 4 charge modulation and its Fourier transformation found in the Sb surfaces of CsV_3_Sb_5_. In (g), there are two Q_1Q_ peaks in addition to Q_Bragg_ and Q_3Q_. Adapted from [61].

Rotational symmetry breaking without translational symmetry breaking, namely nematicity, is another important issue for understanding unconventional electron liquids [73,74]. For KV_3_Sb_5_, low-temperature STM data above SC *T_c_* at zero field showed that the CDW peak intensities at Q_3Q_ show a *C*_6_ rotation broken feature [38,43,63], as shown in Fig. 6(a) as a simulation of 2 × 2 vector peaks on the surface based on bulk 2 × 2 × 2 CDW. Magnetoresistance measurements in CsV_3_Sb_5_ also reveal the nematic nature of the CDW state persisting into the superconducting phase [40,41], as shown in Fig. 6(b). Therefore, the CDW state is electronically nematic with only *C*_2_ rotation symmetry at low temperature. Note that the *z*-direction-modulated CDW reduces the point group symmetry from *D*_6*h*_ down to *D*_2*h*_ [43,72]. However, from the magnetoresistance data in Fig. 6(c), the onset of electronic nematicity is around 15 to 60 K depending on the magnetic field strength [40]. Hence, the electronic nematic transition seems to be separated from the CDW transition at least in CsV_3_Sb_5_. More than that, the signature of this nematic transition can also be found in μSR, coherent phonon spectroscopy and Raman spectroscopy [42,75,76]. The muon spin relaxation rate has a second feature around *T* = 30 K in addition to the onset of the primary TRSB transition [70]. Optical data performing coherent phonon spectroscopy show that a 3.1-THz peak appears below 30 K–60 K in addition to the 1.3-THz peak coupled to the onset of the CDW and 4.1-THz normal peaks [75,76], as shown in Fig. 6(d). Raman spectroscopy also reveals additional peaks below 30 K [42], as plotted in Fig. 6(e). A similar 40-K transition was also identified from the NMR measurement [77]. Hence, it is highly possible that there is an electronic nematic transition around 30 K–40 K in CsV_3_Sb_5_.

Additionally, STM experiments show an in-plane 1 × 4 charge modulation below 50 K ∼ 60 K [61,62,64], as shown in Fig. 6(f). From the Fourier transform of STM topographs shown in Fig. 6(g), there is one additional CDW peak (Q_1Q_) appearing alongside the structural Bragg peaks (Q_Bragg_) and 2 × 2 CDW peaks (Q_3Q_) [61]. Since similar 1 × 4 charge orders have been widely found in cuprates [78–80], this 1 × 4 charge order has attracted considerable attention. To date, however, bulk measurements such as x-ray scattering and NMR still fail to confirm this 1 × 4 order [81]. As it depends on the cleaved surface environment [42,43,52,67], this 1 × 4 charge order may come from a surface manifestation of the intermediate 30–60 K transition, which is supported by the DFT calculations [66]. On the other hand, we should note that observing diffuse quasi-1D correlations in a system that has three such domains is very challenging in conventional x-ray measurements, which calls for further exploration.

For the mirror symmetry, there is still a lack of conclusive evidence for its existence or absence at low temperatures. For example, the STM data in [38] breaks all the mirror symmetries, while another measurement shows one remaining mirror symmetry in [63]. However, using the knowledge discussed above, the largest point group of AV_3_Sb_5_ at low temperature is *D*_2*h*_. Since ${\cal I}$ is a good symmetry, there are only three possible point groups, *D*_2*h*_, *C*_2*h*_ and *C_i_*, which calls for further experimental investigations to determine the remaining symmetries, especially the bulk sensitive measurements.

## THEORIES AND MODELS

**Figure 7.**
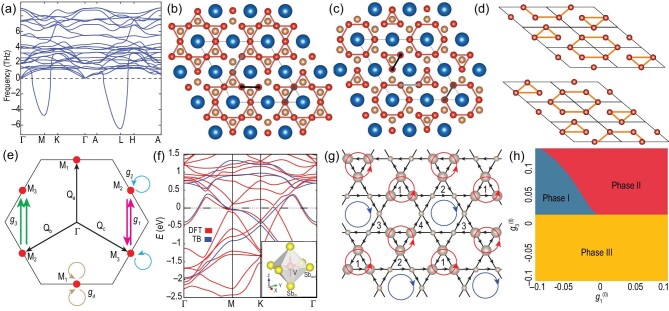
(a) Phonon spectrum calculated for CsV_3_Sb_5_. (b) and (c) Star of David and tri-hexagonal distortions for CsV_3_Sb_5_. Adapted from [52]. (d) 3D structure distortion for AV_3_Sb_5_ with a π shift between the adjacent kagome layers. (e) The low-energy effective theory of three VH points *M*_1–3_ for AV_3_Sb_5_. The arrows denote the scattering processes described by interactions *g*_1–4_. (f) Band structure for the minimal model for CsV_3_Sb_5_. (g) The flux configuration for the chiral flux phase. (h) Renormalization group phase diagram for the effective model. Adapted from [82].

Theoretically, how one models and describes the AV_3_Sb_5_ materials, especially their unconventional CDW states, becomes a crucial question. As discussed above, DFT calculations qualitatively agree with the electronic structures of AV_3_Sb_5_ from ARPES measurements. Therefore, DFT calculations could provide a reasonable starting point for the understanding of AV_3_Sb_5_. Since the structural transition is found to play a vital role in the CDW formation, the most stable structural distortion can be probed by DFT. For example, in CsV_3_Sb_5_, phonon dispersion relations are calculated from the *ab initio* DFT calculations shown in Fig. 7(a) [83]. From the phonon modes, one finds that there are two negative energy soft modes around the M and L points. The structural instabilities led by these soft modes, the ‘Star of David’ (SoD) and ‘tri-hexagonal’ (TrH) structure configurations are proposed to be the likely candidates for CDW structures [52,75,83], as illustrated in Fig. 7(b) and (c). Note that TrH is also named the ‘inverse Star of David’ in the literature. Based on XRD data, STM and quantum oscillation measurements, the TrH state is suggested to be the promising ground-state configuration below *T*_CDW_ in a single-layer model. To accomplish the 2 × 2 × 2 structure modulation, a π shift between the adjacent kagome layer TrH distortions is needed [43,75], as illustrated in Fig. 7(d). On the other hand, recent studies have suggested that the average structure shows signatures of both TrH and SoD structures in the staggered layer sequence [52], which calls for further investigation.

Beyond the structural transition, a model that captures the electronic properties of AV_3_Sb_5_ is important. DFT calculations and ARPES measurements show that multiple bands cross the Fermi level [30,31]. As discussed above, the in-plane Sb *p_z_* orbital forms one electron pocket around the Γ point and the V *d* orbitals form multiple FSs around the M points, as illustrated in Fig. 7(e) [84]. It is very difficult to capture such a complicated Fermi surface topography in a simplified tight-binding model. However, the essential electronic structure of AV_3_Sb_5_ is widely believed to be dominated by the quasiparticles around the VH points based on the following facts. First, the VH points are very close to the Fermi level as obtained from DFT calculations and ARPES measurements [31,50,51]. Second, the quasiparticle interference spectrum shows that the dominant scattering momenta are 3Q (Q_*a*_, Q_*b*_, Q_*c*_) related to three M points as well as the Γ-point FS-induced *q*_1_ scattering [61,62], as illustrated in Fig. 7(e). Finally, the CDW gap size is at maximum around the VH points while its vanishes at the Γ pocket [50,51]. Therefore, a minimal model capturing the VH points and Γ-point FS could faithfully describe the physics behind AV_3_Sb_5_ [52]. Following this spirit, a minimal four-band model based on the V local $d_{X^2-Y^2}$ orbital and in-plane Sb *p_z_* orbital is proposed, as shown in Fig. 7(f) [85]. And the V local $d_{X^2-Y^2}$ orbital model is adiabatically connected to the nearest-neighbor tight-binding model in the kagome lattice. This model provides a solid ground for further theoretical investigation.

The most intriguing property of the AV_3_Sb_5_ CDW is its TRSB. However, neutron scattering, NMR and μSR experiments have already ruled out the possibility of long-range magnetic order with conventional moments in the resolution of the measurements [30,37,69,70]. This feature is reminiscent of long-discussed flux phases in condensed matter, such as the Haldane model on the honeycomb lattice [86]. Moreover, the flux phases breaking TRSB are also widely discussed in cuprate superconductors after the seminal study by Affleck and Marston in t–J models [87,88]. Generalizing this idea, Varma [89] proposed a loop-current phase formed in the Cu-O triangles and Chakravarty *et al.* [90] proposed the d-density wave state with staggered flux in Cu square plaquettes. Both states break the time-reversal symmetry and are candidates for the pseudogap in cuprates [57,89–92].

For kagome lattices and other hexagonal lattices, the 3Q electronic instabilities at VH filling have been widely discussed [26,28,29,93–99], including chiral spin density wave order, charge bond orders, intra-unit cell CDW and *d*+i*d* SC, etc. Based on the minimal model and the 3Q electronic instabilities, several TRSB flux states have been proposed to explain the TRSB. The most promising candidate is the chiral flux phase among the 18 flux classes [72,82,84,100,101]. In this chiral flux state shown in Fig. 7(g), there are two special flux loops. The two anti-clockwise triangle current flux loops (red circles) form a honeycomb lattice and the clockwise hexagonal current flux (blue circle) forms a triangular lattice. The charge order of the chiral flux phase coincides with 2 × 2 charge order and the TrH lattice configuration [84].

Microscopically, how to stabilize the flux state is still under debate. Starting from the VH points, the low-energy effective theory of AV_3_Sb_5_ can be constructed by projection [72,82], as illustrated in Fig. 7(g). Using the parquet renormalization group, various leading and subleading instabilities have been determined, including superconductivity, charge order, orbital moment and spin density waves [82]. For example, a renormalization group phase diagram is shown in Fig. 7(h) when the bare interaction is *g*_2_ > 0. There are three possible phases, I, II and III. Although both the leading and subleading instabilities have been discussed in this work, we only focus on the leading one. Among these three phases, the leading instability of phase II is the ‘imaginary charge-density wave’, which is the low-energy version of the flux phase. In this case, we find that the TRSB phase can be stabilized if the bare interaction *g*_1_ is negative and *g*_2_, *g*_3_, *g*_4_ are positive. But how to achieve attractive interactions needs to be further explored [82]. An extended Hubbard model with on-site Hubbard interaction U and nearest-neighbor Coulomb interaction V is also proposed to stabilize the TRSB order [84,100]. However, the TRSB order has not been found in the realistic parameter region in this type of model. Phenomenologically, the various Ginzburg Landau theory approaches have also been discussed to describe the TRSB phases [82,100,101].

## SUPERCONDUCTIVITY

Superconductivity remains an important property of AV_3_Sb_5_ materials. We focus on discussing the superconducting mechanism and pairing symmetry. Whether an SC is driven by electron-phonon coupling, or unconventionally driven by electron-electron correlation, is the central issue we need to address. To find clues for this hard-core question, we first focus on the superconducting pairing symmetries of AV_3_Sb_5_. Since the inversion symmetry ${\cal I}$ is always a good symmetry for AV_3_Sb_5_, as found in SHG measurements [70], the spin-singlet pairing and spin-triplet pairing must be separated.

**Figure 8.**
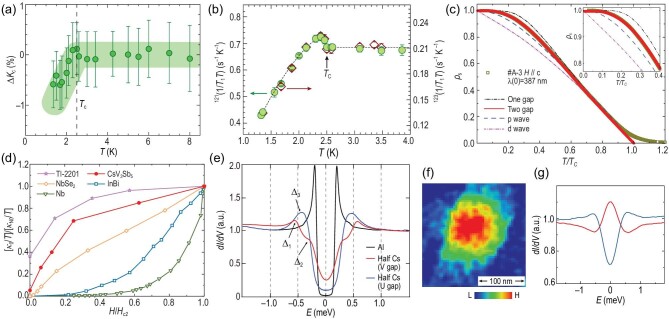
(a) Temperature dependence of the Knight shift Δ*K* of ^121^Sb for CsV_3_Sb_5_ with *H*//*c*. Adapted from [102]. (b) Temperature dependence of ^121^(1/*T*_1_*T*) (left axis) and ^123^(1/*T*_1_*T*) (right axis). A Hebel-Slichter coherence peak appears just below *T_c_* for CsV_3_Sb_5_. Adapted from [102]. (c) The normalized superfluid density ρ_*s*_ for CsV_3_Sb_5_ as a function of the reduced temperature *T*/*T_c_*. Adapted from [107]. The dash-dot-dot, solid, dashed and dash-dot lines respectively represent fits to models with a single s-wave gap, two s-wave gaps, a p-wave gap and a d-wave gap. The inset is en enlargement of the low-temperature region. (d) The normalized residual linear term κ_0_/*T* of CsV_3_Sb_5_ as a function of *H*/*H*_*c*2_. Similar data for *Nb*, InBi, NbSe_2_ and an overdoped d-wave cuprate superconductor Tl-2201 are shown for comparison. Adapted from [34]. (e) Two kinds of superconducting gap spectra observed on the half-Cs surface for CsV_3_Sb_5_. Adapted from [60]. (f) The *dI*/*dV* map showing a superconducting vortex on the Cs surface for CsV_3_Sb_5_. Adapted from [62]. (g) Tunneling spectra obtained in the vortex core (red) with zero-bias peak and outside the vortex (dark blue). Adapted from [62].

To reveal the pairing properties, multiple experimental techniques have been applied. The first task is to determine whether the Cooper pairs form a singlet or triplet, which can be determined through the temperature-dependent spin susceptibility. From the NMR spectrum shown in Fig. 8(a), one finds that the temperature-dependent *z*-direction Knight shift of ^121^Sb drops below the SC transition *T_c_* in CsV_3_Sb_5_[102]. The Knight shifts in the other two directions also show a similar drop below *T_c_* [102]. Therefore, the ground state of AV_3_Sb_5_ belongs to a spin-singlet SC. Additionally, the μSR measurements fail to detect any additional TRSB signals below *T_c_*, compared to the distinct increase in the Sr_2_RuO_4_ SC [103], suggesting a time-reversal invariant superconducting order parameter [69,70,104]. Therefore, the SC order parameter of AV_3_Sb_5_ belongs to the time-reversal preserved spin singlet.

The superconducting gap structure can also provide information about the pairing symmetry. A Hebel-Slichter coherence peak appears just below *T_c_* in CsV_3_Sb_5_ from the spin-lattice relaxation measurement of the ^121/123^Sb nuclear quadrupole resonance [102], as shown in Fig. 8(b). This coherence peak is widely known as a hallmark for a gapped conventional s-wave SC [105,106]. Moreover, an exponential temperature dependence of magnetic penetration depth is found at low temperatures, suggesting a nodeless superconducting gap structure for CsV_3_Sb_5_ [104,107], as shown in Fig. 8(c). No subgap resonance state is found near non-magnetic impurities, while the magnetic impurities destroy the SC quite efficiently from STM measurements [60]. Hence, the SC of AV_3_Sb_5_ is a conventional spin-singlet SC. This feature is also consistent with the weakly correlated nature of AV_3_Sb_5_ and remarkable electron-phonon coupling of the V-derived bands found from ARPES [51].

However, this simple picture is complicated by experimental observation of nodes or deep minima in the superconducting gap. From thermal transport measurements, a finite residual thermal conductivity κ_0_ at *T* → 0 has been found in CsV_3_Sb_5_, which suggests a nodal feature of the pairing order parameter [34,108]. This residual thermal conductivity κ_0_ also shows a similar magnetic field dependence found in a d-wave cuprate, as shown in Fig. 8(d). Additionally, a multiple-gap feature is resolved from the millikelvin STM measurements, as shown in Fig. 8(e). The multi-gap behavior agrees with the multiple FSs revealed from the DFT calculations and the ARPES measurement. Interestingly, in different regions of CsV_3_Sb_5_, both the U-shaped and V-shaped suppression of the density of states have been observed at the Fermi level with a relatively large residual density of state that can hardly be explained by thermal excitations [60,64]. These findings, on the other hand, prefer a superconducting gap with nodes.

This leads to a seeming dichotomy between gapless excitations in the SC state and a conventionally gapped s-wave SC for AV_3_Sb_5_. However, if we take the TRSB normal states into account, the gapless excitations arise within a fully opened superconducting gap [85]. There are two key discrete symmetries in SCs to guarantee the presence of Cooper pairing: time-reversal ${\cal T}$ and inversion symmetry ${\cal I}$ [109–111]. For the even-parity spin-singlet pairing formed by (*c*_*k*, ↑_*c*_−*k*, ↓_ − *c*_*k*, ↓_*c*_−*k*, ↑_), the system at least contains time-reversal symmetry ${\cal T}$ because ${\cal T}$ maps a |*k*, ↑〉 state to a | −*k*, ↓〉 state. Similarly, the odd-parity, spin-triplet pairing needs inversion symmetry ${\cal I}$ owing to the fact that *I* maps a |*k*, ↑〉 state to a | −*k*, ↑〉 state. These two symmetry conditions are known as Anderson’s theorem [109–111]. For AV_3_Sb_5_ SC cases, the normal state before the SC transition breaks the ${\cal T}$ symmetry as discussed above. Therefore, the edge modes on CDW domain walls or other places where the TRSB dominates cannot be gapped out by the SC pairing. These gapless excitations could contribute a finite residual thermal conductivity.

Although SC seems to be conventional, the non-trivial band structure of AV_3_Sb_5_ could lead to non-trivial excitations. Based on Fu-Kane’s seminal proposal, if the helical Dirac surface states of a topological insulator are in proximity to an s-wave SC, Majorana zero modes (MZMs) may arise inside the vortex cores of the superconducting Dirac surface states [112]. The proposal has been widely used in Bi_2_Te_3_/NbSe_2_ heterostructures, and in the iron-based SC Fe(Te,Se), (Li_1 − *x*_Fe_*x*_)OHFeSe, etc. [113–121]. Similar to these aforementioned materials, AV_3_Sb_5_ hosts Dirac surface states near the Fermi energy [31] that can open a superconducting gap below T_*c*_. Therefore, MZMs are theorized to emerge inside the vortex core. Using STM, zero-bias states with spatial evolution similar to the zero-bias peaks in Bi_2_Te_3_/NbSe_2_ heterostructures have been resolved in the vortex cores of CsV_3_Sb_5_ [62], as shown in Fig. 8(f) and (g).

**Figure 9.**
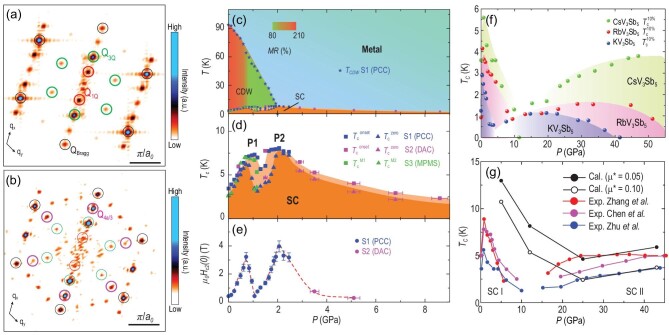
(a) Fourier transformation of atomically resolved STM topography of the Sb surface for CsV_3_Sb_5_. (b) The *dI*/*dV* map at −0.25 meV for CsV_3_Sb_5_ at *T*_electron_ = 300 mK. Comparing to (a), there are additional peaks at *Q*_4/3*a*_. Adapted from [64]. (c) Phase diagram for CsV_3_Sb_5_ with pressure. CDW transition temperature *T*_CDW_ gradually suppressed with increasing pressure. The color inside the CDW represents the magnitude of magnetoresistance measured at 9 T and 10 K. (d) Pressure dependence of superconducting transition temperatures showing two dome behavior. (e) Pressure dependence of the upper critical field at zero temperature. Adapted from [123]. (f) Temperature-pressure phase diagram of AV_3_Sb_5_. Adapted from [127]. (g) Electron-phonon calculated *T_c_* for CsV_3_Sb_5_ and its comparison with experiments. Adapted from [129].

In addition, CsV_3_Sb_5_ may host an intriguing electronic state, known as the pair density wave (PDW), in which the Cooper-pair density modulates spatially at a characteristic wave vector. A low-temperature STM study on CsV_3_Sb_5_ found that both the height of the superconducting coherence peak and the zero-energy gap depth show spatial modulations with a distinct periodicity of 4*a*/3, suggesting a PDW state [64]. In the Fourier transforms of the differential conductance maps taken inside the superconducting gap, six additional Q_4/3*a*_ modulation peaks were found in addition to the 2 × 2 CDW peaks Q_3Q_, 1 × 4 CDW peaks Q_1Q_ and Bragg peaks shown in Fig. 9(a) and (b). Four of these additional Q_4/3*a*_ vectors cannot be obtained by linear combinations of Q_3Q_ and Q_1Q_ peaks, which provides evidence for the PDW in AV_3_Sb_5_ [64].

As the superconductivity in AV_3_Sb_5_ arises within the pre-existing CDW states, exploring the correlation between these two states can help to reveal the underlying physics [122–128]. By applying external pressure to CsV_3_Sb_5_, CDW order becomes destabilized quickly and vanishes at 2 GPa, while the SC state shows a double-peak behavior with a maximum of 8 K around 2 GPa [122,123], as plotted in Fig. 9(c). The competition between the CDW and SC is a common feature of all AV_3_Sb_5_ materials, while the double-peak behavior is clearest in CsV_3_Sb_5_ [127]. Hence, the CDW order highly correlates with the SC in the low-pressure region, known as SC I. By further increasing the pressure, a new SC dome, named SC II, appears for all AV_3_Sb_5_ materials, as shown in Fig. 9(d). A recent DFT calculation with electron-phonon coupling shows that the *T_c_* calculated from the McMillan-Allen-Dynes formula qualitatively agrees with the experimental values obtained above 20 GPa [129], as shown in Fig. 9(e). Hence, the SC-II state at high pressure likely stems from the electron-phonon coupling. However, the *T_c_* calculated based on electron-phonon coupling in the low-pressure range is far above the experimental values, which cannot give rise to a reliable conclusion. The underlying pairing mechanism for AV_3_Sb_5_ needs more experimental exploration and theoretical analysis.

## SUMMARY AND PERSPECTIVE

In this article, we have reviewed the physical properties of the newly discovered kagome materials AV_3_Sb_5_. Owing to tremendous efforts during the past years, we have achieved considerable understanding of AV_3_Sb_5_, which can be summarized as follows.

- AV_3_Sb_5_ is a quasi-2D electronic system with cylindrical Fermi surfaces, where the electronic properties are dominated by the V-Sb kagome layers.
- AV_3_Sb_5_ is a multi-band system with at least four bands crossing the Fermi level. The FS around the Γ point is attributed to the Sb *p_z_* bands, while FSs around the BZ boundary mainly consist of V *d* orbitals. The VH points at the M points play an important role in the unconventional properties of AV_3_Sb_5_.
- Owing to band inversions at M points, AV_3_Sb_5_ is a *Z*_2_ topological metal with unconventional surface states.
- The correlation strength of AV_3_Sb_5_ is weak based on DFT calculations and ARPES measurements.
- AV_3_Sb_5_ undergoes a first-order phase transition into charge density wave order around 80 to 104 K, depending on the A-site cation. Within the kagome layer, the CDW enlarges the unit cell to 2 × 2 accompanied by a *c*-axis modulation.
- There is evidence for the emergence of time-reversal symmetry breaking inside the CDW state. Besides translational symmetry breaking and time-reversal symmetry breaking, inversion symmetry perseveres while *C*_6_ rotation symmetry is broken.
- The superconducting order parameter of the AV_3_Sb_5_ SC is a spin singlet with *T_c_* around 1–3 K, depending on the A-site cation. The SC appears to be a conventional s-wave with unconventional excitations inside the vortex core. The CDW order is intertwined with the SC in an unconventional way, inducing multiple SC domes under pressure.

The discovery of the AV_3_Sb_5_ SC opens a new route towards realizing unconventional orders within 2D kagome metals, which brings us a new platform to investigate the interplay between correlation, topology and geometric frustration. We hope that this review provides a broad picture of the recent progress on AV_3_Sb_5_ kagome materials and stimulates new research frontiers within kagome-related physics.
